# Effect of Surface Roughness on Aggregation of Polypeptide Chains: A Monte Carlo Study

**DOI:** 10.3390/biom11040596

**Published:** 2021-04-18

**Authors:** Nguyen Truong Co, Mai Suan Li

**Affiliations:** 1 Institute of Physics, Polish Academy of Sciences, Al. Lotnikow 32/46, 02-668 Warsaw, Poland; ntco@ifpan.edu.pl; 2 Institute for Computational Science and Technology, SBI Building, Quang Trung Software City, Tan Chanh Hiep Ward, District 12, Ho Chi Minh City 700000, Vietnam

**Keywords:** protein aggregation, aggregation of polypeptide chains, fibril formation, neurodegenerative diseases, surface roughness, lattice model

## Abstract

The self-assembly of amyloidogenic peptides and proteins into fibrillar structures has been intensively studied for several decades, because it seems to be associated with a number of neurodegenerative diseases, such as Alzheimer’s and Parkinson’s disease. Therefore, understanding the molecular mechanisms of this phenomenon is important for identifying an effective therapy for the corresponding diseases. Protein aggregation in living organisms very often takes place on surfaces like membranes and the impact of a surface on this process depends not only on the surface chemistry but also on its topology. Our goal was to develop a simple lattice model for studying the role of surface roughness in the aggregation kinetics of polypeptide chains and the morphology of aggregates. We showed that, consistent with the experiment, an increase in roughness slows down the fibril formation, and this process becomes inhibited at a very highly level of roughness. We predicted a subtle catalytic effect that a slightly rough surface promotes the self-assembly of polypeptide chains but does not delay it. This effect occurs when the interaction between the surface and polypeptide chains is moderate and can be explained by taking into account the competition between energy and entropy factors.

## 1. Introduction

The self-assembly of proteins into aggregates of various morphologies is probably one of the main causes of chronic neurodegenerative diseases such as Alzheimer’s, Parkinson’s and Huntington’s disease [1,2,3]. Therefore, understanding the mechanisms of protein aggregation plays an important role in discovering effective therapies to treat these diseases. Protein aggregation can occur in solution as well as in complex environments, including various surrounding objects such as cell membranes, DNA, sugars, other biological compounds and industrial artificial surfaces which require the careful study of the effect of different surfaces on the process [4,5,6,7,8]. In general, the impact of foreign surfaces on the protein aggregation process displays complicated behaviors which depend on the type of surfaces, proteins and experiment conditions [9,10,11,12]. Lipid membranes were reported to play the role of a template to accelerate the aggregation of different amyloidogenic peptides [13,14,15,16]. Mica and glass facilitated the fibril formation of the fragment Aβ_18-22_ [17] and α-synuclein [18] on their surfaces, respectively. However, the surfaces of polymeric nanoparticles slow the self-assembly of IAPP [19], while the protein-coated surfaces of graphene oxide showed a strong inhibition effect for the fibrillogenesis of peptide Aβ_42_ [20]. Carbon nanotubes [21,22] and nanoparticles [23,24] can either accelerate or slow down aggregation depending on the experimental conditions and aggregation agents. Furthermore, the fibril structure of amyloid peptide GAV-9 favors a “stand up” motif on the hydrophilic mica surface, but greatly prefers the “lie down” position on a hydrophobic HOPG (highly oriented pyrolytic graphite) plain [25,26]. Surfaces can also control the peptide aggregation kinetics as well as their morphologies through surface topologies [27,28] or surface roughness [29,30]. Furthermore, many computational investigations have been conducted to explore the role of smooth surfaces and membrane on protein aggregation using coarse-grained [31,32] and all-atom models [33].

Combining a two-state peptide model [34] and experiment on the self-assembly of Aβ_42_ and α-synuclein in the presence of different types of surfaces, Vacha et al. [35] have shown that weakly absorbing surfaces retard proteins aggregation, while strongly absorbing surfaces enhance the process. This implies that the aggregation is modulated by interactions between polypeptide chains and foreign surfaces. When proteins are absorbed on the surface, their movement becomes more limited compared to the three-dimensional case, and this can lead to an increase or decrease in the fibril formation rate depending on the experimental conditions [36]. A weakly absorbing surface reduces the concentration of proteins in the bulk, which reduces the probability of the formation of critical nuclei there [35]. On the other hand, the interactions between surface and polypeptide chains are not strong enough to maintain a sufficient number of chains on the surface to accelerate the aggregation process. Competition between these factors leads to a decrease in the overall rate of protein aggregation on weakly absorbing surfaces [37]. For highly absorbent surfaces, a large amount of proteins is absorbed on the surface, catalyzing the formation of critical nuclei, which accelerates the fibril formation [17]. However, very strong absorption will impede the process due to the hindrance of diffusion and sampling of peptides on the plain [36].

Combining polymer coating, argon plasma treatment and thermal annealing techniques, Shezad et al. [29] were successful in the production of polystyrene surfaces with roughness of varying degrees. They showed that the aggregation rate of Aβ42 (beta amyloid peptide of 42 residues) decreases with increasing surface roughness, and very rough surfaces even block fibril growth. Using various experimental methods, it was shown that a the rough surface restricts the two-dimensional diffusion of peptides, which slows down the surface-mediated formation of fibrillar species [29].

To our best knowledge, theoretical studies of the impact of surface roughness on protein aggregation have not been conducted. In this paper, we develop simple lattice models in which rough surfaces were created by randomly distributed balls on smooth planes and performed Monte Carlo simulations to explain the experimentally observed phenomena. More importantly, we predicted that for moderate particle–surface interactions, slightly rough surfaces can accelerate the fibril formation rather than slow it down.

## 2. Materials and Methods

### 2.1. Lattice Models in Bulk

Since the fibril formation time of proteins varies from hours to months, its assessment using all-atom or even off-lattice coarse-grained models is not possible within the existing computational capabilities. The problem becomes harder in the presence of rough surfaces that slow down this process. Therefore, we developed simple lattice models that allowed us to estimate the fibril formation rate with a reasonable amount of computational time (our models are simpler than other coarse-grained models [38,39,40,41]). These models are an extension of lattice models that were successfully used by our group to simulate the kinetics of fibril formation of polypeptide chains in the absence of surfaces [42]. Despite their simplicity the lattice models correctly captured the dependence of fibril formation time on the hydrophobicity, charge and population of the so called fibril prone state N* in monomeric state [19]. They were also useful in studying the mechanism of heat-induced amyloid fibril degradation [43], the role of crowders in fibrillation [6] and assessing the size of critical nuclei [44].

**Figure 1:**
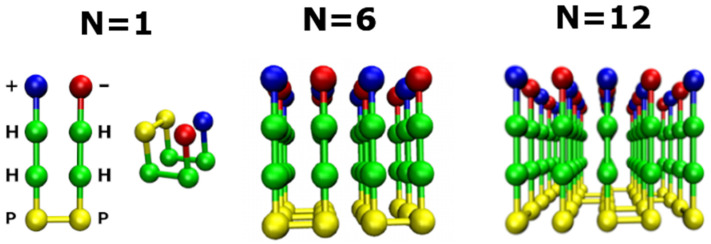
(Left) Single peptide of 8 beads that are **+HHPPHH-** in N* conformation. Red, green, yellow and blue refer to amino acids **−, H, P, +**, respectively. Native structure of 1 peptide chain is showed next to N*. Fibril structure of the systems of N = 6 and N = 12 chains in the lattice model.

In our model, polypeptide chains, which are confined to a discrete cubic lattice, consist of *M* = 8 beads, designated as + HHPPHH−, respectively (Figure 1, left), where +, − represent positively and negatively charged residues, and H and P stand for hydrophobic and polar residues, respectively. Interactions include interactions of beads in the same chain (intra-contact) and interactions of beads from different chains (inter-contact). The intra-chain interaction between two beads is counted if they are not successive in sequence and the distance between them is equal to the lattice spacing *a*. We take into account only the nearest neighbor inter-chain interaction, which occurs when the distance between two interacting beads is equal to *a*. Then, the total energy of the system consisting of *N* polypeptide chains, *E*, is determined by the first two terms of the following expression:

(1) $$\begin{array}{c} E=\sum_{l=1}^{N}\sum_{i<j}^{M}e_{sl(i)sl(j)}\delta (r_{ij}-a)+\sum_{m<l}^{N}\sum_{i,j}^{M}e_{sl(i)sm(j)}\delta (r_{ij}-a)+\sum_{m=}^{N}\sum_{i=1}^{M}\sum_{k=1}^{Ns}e^{vs}{}_{k,sm(i)}\delta (r_{ik}-a) \end{array}.$$

where *r_ij_* is the distance between amino acids *i* and *j*, in simulations *a* is assigned Equal (1), *sm (i)* denotes the amino acid *i*^th^ in the *m*^th^ peptide chain. The delta-function *δ*(x) is 1 at x = 0 and 0 otherwise. The first and second terms in Equation (1) describe the intra- and inter-chain interactions, respectively. The pair interactions between beads (or “force field”) *e_ij_* are given in Table 1. The third term describes the interaction between polypeptide chains and surface (see below). In our models, 20 amino acids were divided into hydrophobic, hydrophilic, negatively charged and positively charged groups. As in our previous works [42,45], their interaction energies shown in Table 1 and Table 2 were basically selected based on the statistical potentials that were obtained by Betancourt and Thirumalai [46].

By Monte Carlo simulations [19,42] (see also below) we obtained the fibril structures for the systems with N = 6 and 12 peptides (Figure 1).

### 2.2. Lattice Models with Surfaces

#### 2.2.1. Smooth Surface

We modeled a smooth surface as a systems of beads regularly located at points of a two-dimensional square lattice. The lattice spacing of the surface is the same as in bulk. The surface beads are kept fixed during the simulation, and the interaction between them is not taken into account. The chemical properties of surfaces are characterized by their interaction with polypeptide chains. We examined two types of surfaces: hydrophilic and hydrophobic surfaces, which consist of hydrophobic and hydrophilic beads, respectively. Surface beads are designated as Ps and Hs for the hydrophilic and hydrophobic surfaces, respectively.

Denoting the interaction energy between the bead *i* of a polypeptide chain and the surface bead *j* as *e*^νs^_i,j_ (ν can be p for hydrophilic surface and h for hydrophobic surface), the interaction energy between the polypeptide chains and the surface is given by the third term in Equation (1). Here, *N*s is the total number of sites of the surface area. The *e^ν^*^s^_i,j_ values are shown in Table 2. Here, we chose the interaction between surface beads and polypeptide beads to be the same as the interaction between the beads of polypeptide chains. However, in order to explore the dependence of fibril formation kinetics on the strength of interaction with surfaces, we varied the interaction between the hydrophobic beads of the polypeptide chains and the surface ε^hs^ and the interaction between hydrophilic beads of polypeptide chains and surface ε^ps^.

**Figure 2:**
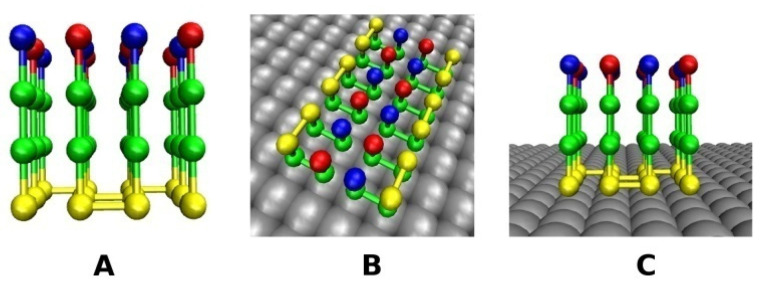

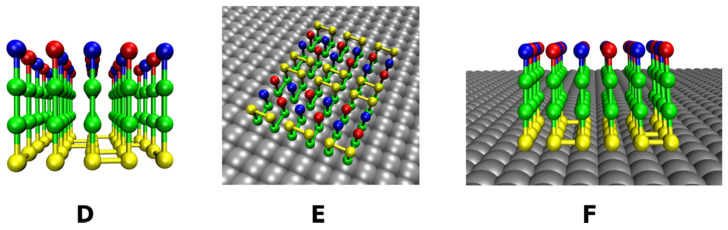
Fibril structure of six polypeptide chains in bulk (**A**), on hydrophobic surface (**B**) and hydrophilic surface (**C**). Fibril structure of 12 polypeptide chains in bulk (**D**), on hydrophobic surface (**E**) and hydrophilic surface (**F**).

Depending on the surface, fibrils may have different morphologies. In our model, for N = 6 and 12 chains, the fibril structure on the hydrophilic surface has the same shape (Figure 2C,F) as in bulk (Figure 2A,D), but on the hydrophobic surface all the chains adopt the monomer native structure (Figure 1) in the fibril state due to strong hydrophobic interactions between the chains and the surface and the short peptides in our model (Figure 2B,E). In other words, the fibril-prone conformation N* coincides with the compact native conformation. Thus, even in the simple model, we showed that the surface can change the morphology of fibrils.

#### 2.2.2. Rough Surfaces

**Figure 3:**
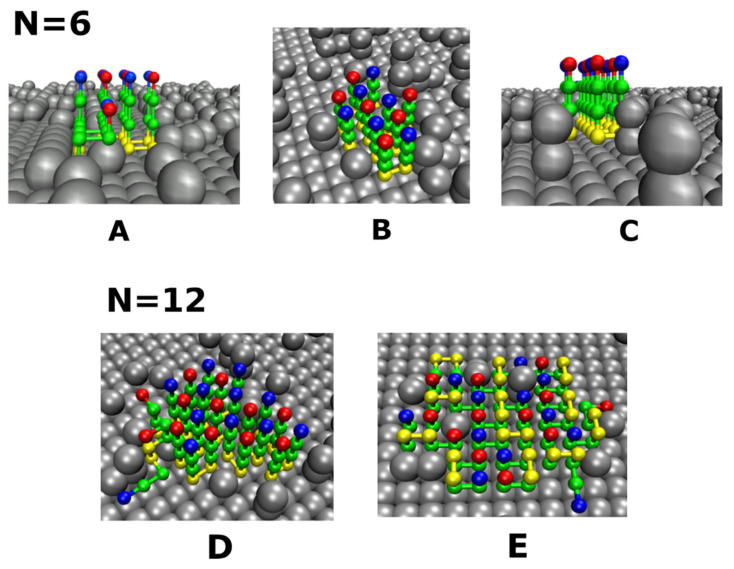
Typical fibril-like structures for the system of N = 6 chains on rough hydrophilic surfaces with single balls (S-surface) (**A**), the combination of single and double balls (DS-surface) (**B**), and double balls (D-surface) (**C**). The fibril-like configuration of 12 chains on the hydrophilic DS- surface (**D**), and hydrophobic DS-surface (**E**).

To build a rough surface, we randomly placed identical balls on a smooth surface. These balls should be hydrophobic and hydrophilic for rough hydrophobic and hydrophilic surfaces, respectively. Each ball has the same size and occupies one lattice site similar as the chain’s beads; however, in order to facilitate the visualization, we exhibited them in larger size (Figure 2 and Figure 3). The roughness depends not only on the concentration of randomly distributed balls, but also on how they are arranged. In this paper, we considered 3 types of arrangement: single balls (S-point), double balls (D-point) and the equal mix of S-point and D-point called DS-point (Figure 3); therefore, we have S-surface, D-surface and DS-surface. In the first case, at each position, only one ball was positioned at *a* distance of the grid pitch *a* from the surface (Figure 3A). In the case of a D-surface, two rigidly connected balls were placed perpendicular to the surface and the distance between the higher ball and the surface is equal to 2*a* (Figure 3C). Finally, the DS-surface comprises an equal number of S-ball and D-ball randomly distributed on a smooth surface (Figure 3B).

### 2.3. Definition of Surface Roughness

We define the distance (or height) from the ball *i* to the surface as *h_i_*. Then, *h_i_* takes the values 1, 2 and both values for the S-, D- and DS-surface, respectively. For surface point *j*, without ball, *h_j_* = 0. The average distance between the surface and randomly distributed balls, $\bar{h}$, is determined by the following formula:

(2) $$\bar{h}=\frac{\sum_{i=1}^{N_{s}}h_{i}}{N_{s}},$$

where *N_s_* is the number of points of the surface. Then, the degree of surface roughness (or roughness) is the standard deviation of *h* and is defined as follows:

(3) $$\Theta =\sqrt{\frac{1}{N_{s}}\sum_{i=1}^{N_{s}}{\left(h_{i}-\bar{h}\right)}^{2}}.$$

Defining Ω as the percentage of points at which single and double balls are placed to create surface roughness, we have:

(4) $$\Omega =\frac{N_{b}}{N_{s}}$$

where *N_b_* is the number of points with balls.

### 2.4. Monte Carlo Simulation

The concentration of chains that were enclosed in a cubic simulation box was brought to about 6 μM (about 10 times higher than the experimental concentration) for all simulated systems. Although the lattice model is simple, calculating the aggregation time of many polypeptide chains requires an enormous amount of computational resources, especially in the presence of rough surfaces. Therefore, we used a high concentration of peptides to accelerate the aggregation process. We applied the periodic boundary conditions to minimize finite size effects. The Monte Carlo (MC) algorithm was utilized to determine the dynamics of peptide chains. MC moves involved local and global moves. The local move may be tail rotation, corner flip, or crankshaft. The global move is either the rotation of a peptide at a 90-degree angle around a randomly selected coordinate axis, or the translation of a peptide on *a* in a random direction [19,42]. The probability ratio between global and local moves was chosen as 1:9. The combination of the local move and global move is the Monte Carlo step (MCS), which is a time unit in the lattice model. Although the equivalence between real time and MCS remains controversial, lattice models have been helpful in understanding the mechanisms of protein aggregation in the bulk [19,43,45] and crowded environment [6]. Note that the fibril structures with the lowest energy obtained using MC simulations are shown in Figure 1, Figure 2 and Figure 3.

It should be noted that we used our homemade programs written to study the self-assembly of polypeptide chains in lattice models. They have been used and developed for different problems since 2008 [42]. We use Fortran 90 and Monte Carlo dynamics. Our code is not parallel and it took about 48 h of CPU on an Intel Xeon E5-2680v3 2.50 GHz, for example, to simulate a trajectory of 12 chains that aggregate on a surface with a roughness of Θ = 0.1. For each data point, we have to average over 150 MC trajectories, which takes a lot of computational time. Therefore, we have to run on the supercomputer TASK located in Gdansk, Poland.

### 2.5. Aggregation Time τ_agg_ and Fibril Formation Time τ_fib_

The fibril formation time τ_fib_ is the number of MCS required to reach the fibril structure from random initial configurations of the peptide chains. In a simulation with many MC trajectories, τ_fib_ is defined as the average value of the first passage times. In the case of smooth surfaces, where the fibril formation is relatively fast, we can estimate τ_fib_ from many MC trajectories. However, for rough surfaces, the fibril formation is so slow that even in simple lattice models, it is not feasible to obtain τ_fib_ within a reasonable amount of time. Therefore, instead of τ_fib_, we introduced the aggregation τ_agg_, which is the first passage time for acquiring a structure that has 80% fibril contacts. For each roughness degree, we generated 10–15 random surface profiles and conducted 15–20 MC runs per profile. Thus, for a given roughness, τ_agg_ was obtained by averaging over 150 (10 × 15) trajectories.

## 3. Results and Discussion

### 3.1. Effect of Smooth Surface on Aggregation Time τ_agg_

#### 3.1.1. Hydrophilic Surfaces

We first examined the effect of smooth surfaces on the aggregation process of system N = 6 polypeptide chains at various temperatures. To estimate τ_agg_, MC simulations were conducted for systems of N = 6, initially random peptides in the cubic box. The number of MC steps to achieve a structure that has 80% fibril contacts was defined as the first passage time. For each parameter set, 100 MC runs were performed and τ_agg_ was the average value of the 100 first passage times.

**Figure 4:**
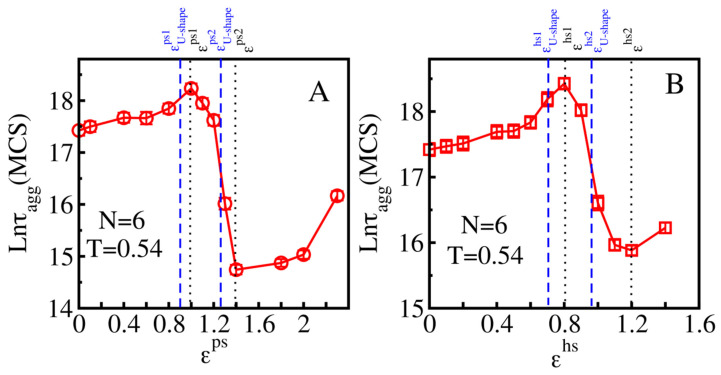

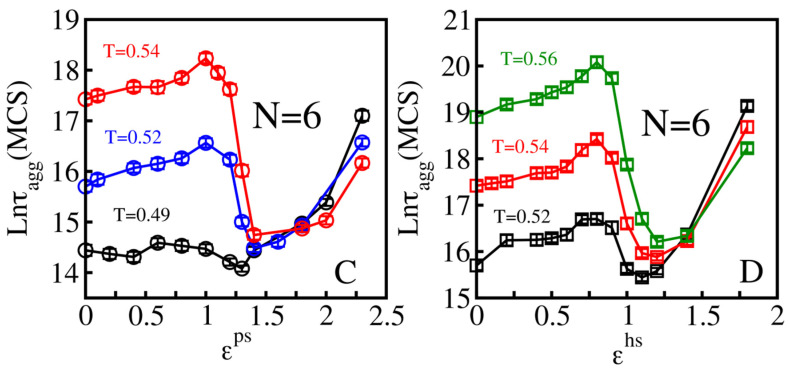
Dependence of lnτ_agg_ on the interaction of the systems consisting of N = 6 polypeptide chains for hydrophilic (**A**,**C**) and hydrophobic (**B**,**D**) smooth surfaces. The upper row exhibits the simulation at T = 0.54, the values ε^ps1^ = 1.0, ε^ps2^ = 1.4, ε^hs1^ = 0.8 and ε^hs2^ = 1.2 (black dashed lines) separate the three interaction regions corresponding to the three types of surfaces. Between $\varepsilon_{U-\mathrm{shape}}^{\mathrm{ps}1}$ = 0.9 and $\varepsilon_{U-\mathrm{shape}}^{\mathrm{ps}2}$ = 1.25 (blue dashed lines), we observed the U-shape dependence of lnτ_agg_ on the roughness degrees Θ for an S-point hydrophilic surface. A similar behavior occurs for a hydrophobic surface between $\varepsilon_{U-\mathrm{shape}}^{\mathrm{hs}1}$ = 0.7 and $\varepsilon_{U-\mathrm{shape}}^{\mathrm{hs}2}$ = 0.95 (blue dashed lines). The notations “ps” and “hs” refers to a hydrophilic and hydrophobic surface, respectively. The bottom row also shows correlation of lnτ_agg_ and interaction parameters ε^ps^ of hydrophilic (**C**) and ε^hs^ hydrophobic (**D**) smooth surfaces for various temperatures and wider ranges of the interaction values. The bottom row shows the dependence of ln*τ*_agg_ on the interaction parameter *ε*^ps^ of hydrophilic (**C**) and ε^hs^ of hydrophobic (**D**) smooth surfaces for various temperatures. Error bars are lower than data symbols.

For a hydrophilic smooth surface of simulation, we varied the interaction between hydrophilic beads of peptides and the surface ε^ps^ in the range (0–2.4). The dependence of τ_agg_ on ε^ps^ is not monotonic (Figure 4A). At T = 0.54, in the weak peptide–surface interaction regime with ε^ps^ varying from 0.1 to 1.0, aggregation is slowed by the surface. This is due to the fact that a weakly adsorbing surface cannot serve as a good template for the initiation of aggregation, and interplay between the formation of an aggregate in the bulk and on the surface slows down self-assembly. As the peptide–surface interaction increases (1.0 < ε^ps^ ≤ 1.4), the surface becomes a good catalytic center for nucleation, which accelerates aggregation (Figure 4A). Our results are consistent with Vacha et al. [35] who reported that weakly absorbing surfaces increase the aggregation time, while strongly absorbing surfaces catalyze the process.

A further increase in the peptide–surface interaction (ε^ps^ > 1.4) again slows down aggregation again (lnτ_agg_ increases from 14.74 to 16.16), but the process remains faster than in the bulk. This increase was not studied by other groups previously and this can be explained by the fact that a strong binding to the interface reduces conformational entropy, which complicates the fibril formation. In other words, strong absorption significantly restricts the mobility monomers on the surface leading to retarded aggregation. As evident from Figure 4A, smooth hydrophilic surfaces have obstacle aggregation for ε^ps^ ≥ 1.4. However, with a very strong peptide–surface interaction, aggregation occurs more slowly than in bulk, as we can see in Figure 4C for T = 0.49 and T = 0.52.

Higher temperatures enhance the flexibility of peptides, leading to a slower self-assembly in the bulk and on the surface (Figure 4C). On the other hand, they can prevent trapping of polypeptide chains on a strongly absorbing surface, which explains why at ε^ps^≥ 1.8 aggregation is accelerated at higher temperatures (Figure 4C). A similar effect was seen for hydrophobic surfaces with ε^hs^ ≥ 1.4 (Figure 4D).

#### 3.1.2. Hydrophobic Surfaces

For T = 0.54, a similar dependence of the aggregation time on the interaction of polypeptide chains with a smooth hydrophobic interface was obtained for ε^hs^, which varies in (0; 1.4) interval (Figure 4B). On a weakly absorbing hydrophobic surface (0.1 < ε^hs^ < 0.8), peptide self-assembly was attenuated with lnτ_agg_ increasing from 17.42 to 18.43. For ε^hs^ in the [0.8;1.2] range, aggregation became enhanced with an increase in the peptide–surface interaction and lnτ_agg_ decreased from 18.43 to 15.88. Finally, lnτ_agg_ leveled from 15.88 to 16.22 as ε^hs^ changed from 1.2 to 1.4.

As in the case of hydrophilic surfaces, the dependence of the aggregation rate on ε^hs^ can be rationalized by the interplay between the peptide–surface interaction and the entropy loss due to the reduction in spatial dimensionality from 3 in bulk to 2 on surface. For a weakly absorbing surface, aggregation takes place mainly in bulk, whereas in the opposite case, self-assembly predominately occurs on the surface. Extended calculations for different temperatures and a wider range of ε^hs^ have been shown in Figure 4D. Higher temperatures can support the aggregation process on very strongly absorbing surfaces (ε^hs^ > 1.4) by promoting peptides’ mobility (Figure 4D). Overall, the three modes of dependence of lnτ_agg_ on ε^hs^ remain unchanged for other temperatures including T = 0.52 and T = 0.56. 

Aggregation in the presence of weakly absorbing surfaces happen dominantly in bulk, therefore the variation of lnτ_agg_ in the simulation time was revealed to be rather similar for both hydrophilic (lnτ_agg_ change from 17.49 to 18.23) and hydrophobic cases (lnτ_agg_ varies in range of [18.47 to 18.42]). For stronger attractive surfaces, the catalyzing (ε^hs^ > 0.8) and retardation effects (ε^hs^ > 1.2) of hydrophobic surfaces happened earlier compared with hydrophilic surfaces (ε^ps^ > 1 and ε^hs^ > 1.4 for catalyzing and retardation effects, respectively). It is not clear whether this observation is always true or sequence dependent. One of the possible reasons for the difference is that the studied sequence has four hydrophobic residues, while the number of polar residues is two.

#### 3.1.3. Three Classes of Surfaces

Based on the aggregation time and the peptide–surface interaction characterized by either the parameter ε^ps^ or ε^hs^, we divided the surfaces into three classes: weakly, medium and strongly absorbing surfaces. The weakly absorbing surface should have ε^ps^ < ε^ps1^ or ε^hs^ < ε^hs1^, where ε^ps1^and ε^hs1^ are the values below which the aggregation time increases with the increasing peptide–surface interaction (Figure 4A,B). For medium absorbing interfaces, ε^ps1^ < ε^ps^< ε ^ps2^ for hydrophilic surfaces and ε^hs1^ < ε^hs^ < ε^hs2^ for hydrophobic surfaces, where ε^ps2^ and ε^hs2^ correspond to the minimum in Figure 4A,B. If the interaction exceeds the minimum values, ε^ps2^ and ε^hs2^, the corresponding surfaces are classified as strongly absorbing surfaces. In the weakly absorbing surface case, the peptide–surface interaction is weak and the aggregation kinetics is, therefore, driven by entropy. In the opposite case of strongly absorbing surfaces, the self-assembly is an energy-driven process.

The values of ε^ps1,2^ and ε^hs1,2^ that divide the surfaces into three classes depend not only the on chemical properties of surfaces, but also on sequences and the number of polypeptide chains. For N = 6, ε^ps1^ = 0.8 and ε^ps2^ = 1.4 for the hydrophilic surfaces and ε^hs1^ = 0.8 and ε^hs2^ = 1.2 for the hydrophobic surfaces (Figure 4).

In our model, the interaction between absorbing particles and the surface is controlled by the parameters ε^ps^ and ε^hs^ for hydrophilic and hydrophobic surfaces, respectively. In general, by varying these parameters, we can qualitatively capture aggregation on different surfaces including membrane surfaces with different lipid compositions and carbon nanotubes (see also Introduction). Namely, depending on ε^ps^ and ε^hs^, aggregation can be either accelerated or slowed down as has been observed experimentally for various systems. For example, Cabaleiro-Lago et al. reported that hydrophobic single-walled carbon nanotubes slow down the fibril formation of Aβ40 peptide [23], which is consistent with all-atom simulations for the truncated variant Aβ_16-22_ [47]. This experimental observation also agrees with our results obtained for ε^hs^ < ε^hs1^ and ε^hs^ > ε^hs2^, where the interaction with the surface retards the aggregation. 

### 3.2. Effect of Surface Roughness on Fibril Formation: S-Surface

As mentioned above, the chemical properties of surfaces control the fibrillar growth of polypeptide chains in various scenarios. Therefore, in order to understand the role of surface roughness in protein self-assembly, we must systematically study this process in the presence of foreign surfaces not only of varying degrees of roughness but also of different peptide–surface interactions.

#### 3.2.1. Weakly Absorbing Surfaces: Monotonic Dependence of the Aggregation Time on the Surface Roughness

**Figure 5:**
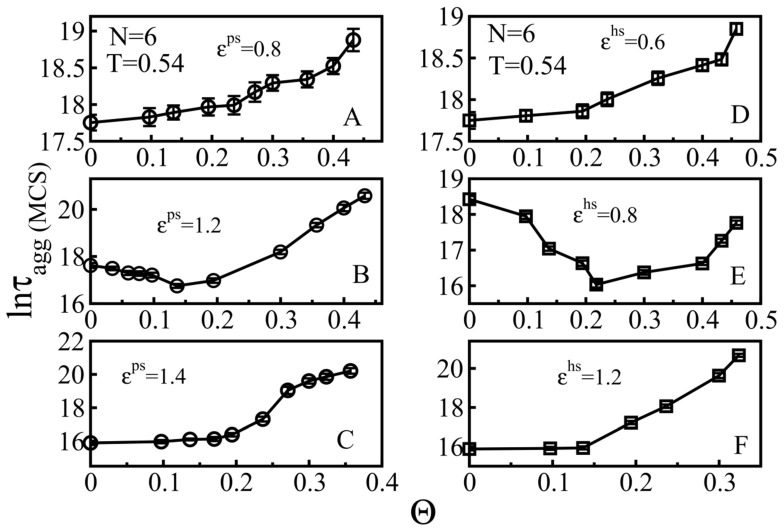
Impact of S-surface roughness on the aggregation of N = 6 peptides at temperature T = 0.54. All graphs on the left column (circular data points) display the dependence of the aggregation time lnτ_agg_ on the rough degree of different hydrophilic surfaces at various values of ε^ps^ = 0.8 (**A**), ε^ps^ = 1.2 (**C**) and ε^ps^ = 1.4 (**E**). Similarly, the dependence ofτ_agg_ on Θ**_S_** for various hydrophobic surfaces is shown on the right graphs (square data points), ε^hs^ = 0.6 (**B**), ε^hs^ = 0.8 (**D**), and ε^hs^ = 1.2 (**F**).

After generating random particles with a given chemical property on a smooth surface, we obtained tunable rough surfaces corresponding to the chosen chemical properties. For weakly attractive surfaces, we chose ε^ps^ = 0.8 and ε^hs^ = 0.6 (Figure 5A,D). Clearly, the higher the degree of roughness, the slower the peptide aggregation process. As can be seen from Figure 5A, where the value of ε^ps^ was set to 0.8 which corresponds to weakly attractive smooth hydrophilic surfaces, as the roughness degrees Θ_S_ changes from 0 to 0.432, lnτ_agg_ increases from 17.75 to 18.87.

A similar result was obtained for a hydrophobic surface (Figure 5D), where ε^hs^ = 0.6, an increase in Θ_S_ from 0 to 0.458 led to an increase in lnτ_agg_ from 17.80 to 18.84.

In the corresponding peptide–surface interaction ranges, the kinetics of aggregation on the rough hydrophilic surfaces is similar to the kinetics on the hydrophobic surfaces (Figure 4 and Figure 5), hinting that there are some emphasized physical principles that govern these phenomena. For weakly attractive surfaces, there is interplay between the peptide assembly in the bulk and on the surface; an increase in roughness improves the ability of the peptides to adhere to the surface, increasing the phase space of the peptides in the bulk, which leads to a decreased aggregation rate (Figure 5A,D). We can call this regime entropy driven. We found that the monotonic increase in τ_agg_ with roughness was observed for ε^ps^ < $\varepsilon_{U-\mathrm{shape}}^{\mathrm{ps}1}$ = 0.9 and ε^hs^ < $\varepsilon_{U-\mathrm{shape}}^{\mathrm{hs}1}$ = 0.7 (blue dashed lines in Figure 4A,B)

#### 3.2.2. Medium Absorbing Surfaces: U-Shape Behavior

In order to study the medium absorbing surfaces, we set ε^ps^ = 1.2 and ε^hs^ = 0.8 and observed a U-shape dependence, namely, that a slightly rough surface accelerates the fibril formation, while a higher roughness degree slows down the peptide self-association (Figure 5B,E). With a sufficiently low roughness, the roughness increases the probability of monomers aggregating on the surface and the attractive force still allows the proteins to diffuse and associate on the surface, forming fibrillar species, which correspond to the acceleration phase in the U-shape effect. However, as the surface roughness exceeds a threshold point at which the combination of the roughness and absorption restricts the mobility of polypeptide chains, a retardation phase of self-assembly occurs. Thus, the U-shape comes from the competition between energy and entropy. We found that this behavior occurred for $\varepsilon_{U-\mathrm{shape}}^{\mathrm{ps}1}$ < ε^ps^ < $\varepsilon_{U-\mathrm{shape}}^{\mathrm{ps}2}$ = 1.25 and $\varepsilon_{U-\mathrm{shape}}^{\mathrm{hs}1}$ < ε^hs^ < $\varepsilon_{U-\mathrm{shape}}^{\mathrm{hs}2}$ = 0.95 (blue dashed lines in Figure 4A,B), for the hydrophilic and hydrophobic surfaces, respectively.

#### 3.2.3. Strongly Absorbing Surfaces: Monotonic Dependence of the Aggregation Time on the Surface Roughness

To investigate this case, we calculated the dependence of the aggregation on the roughness for the parameters ε^ps^ = 1.4 and ε^hs^ = 1.2, which can characterize strongly absorbing hydrophilic and hydrophobic surfaces (Figure 4). In this case, the surface tends to confine the movement of polypeptide chains in two-dimensional space, reducing their mobility. As a result, on very attractive surfaces, there was no acceleration phase at low roughness and the aggregation time rapidly increased with the increasing roughness (Figure 5C,F). The fibril formation becomes practically inhibited for roughness Θ_S_ > 0.356 and 0.323 for hydrophilic (Figure 5C) and hydrophobic (Figure 5F) surfaces, respectively. This result is in qualitative agreement with the experimental result of Shezal et al. [29] for a highly attractive rough surface, as their experiment was conducted at a protein concentration lower than CMC in order to prevent protein aggregation only in bulk. 

We can show that the monotonic increase in Figure 5C,F is possible for ε^ps^ > $\varepsilon_{U-\mathrm{shape}}^{\mathrm{ps}2}$ = 1.25 and ε^hs^ > $\varepsilon_{U-\mathrm{shape}}^{\mathrm{hs}2}$ = 0.95 (blue dashed lines in Figure 4A,B), for the hydrophilic and hydrophobic surfaces, respectively. This behavior appears in the energy-driven regime due to the strong peptide–surface interaction.

### 3.3. Effect of Surface Roughness on Fibril Formation: D-Surface and DS-Surface Do Not Qualitatively Change the Results Obtained for S-Surfaces

Thus far, we considered S-surfaces with a single ball roughness (Figure 3A). In this section, we explore two other cases: a D-surface, where a rough surface is created by randomly placed double balls on a smooth surface (Figure 3C); and a DS-surface, which consists of randomly distributed S-balls and D-balls (Figure 3B). For simplicity, it is assumed that the numbers of S and D balls on the DS-surface are the same.

**Figure 6:**
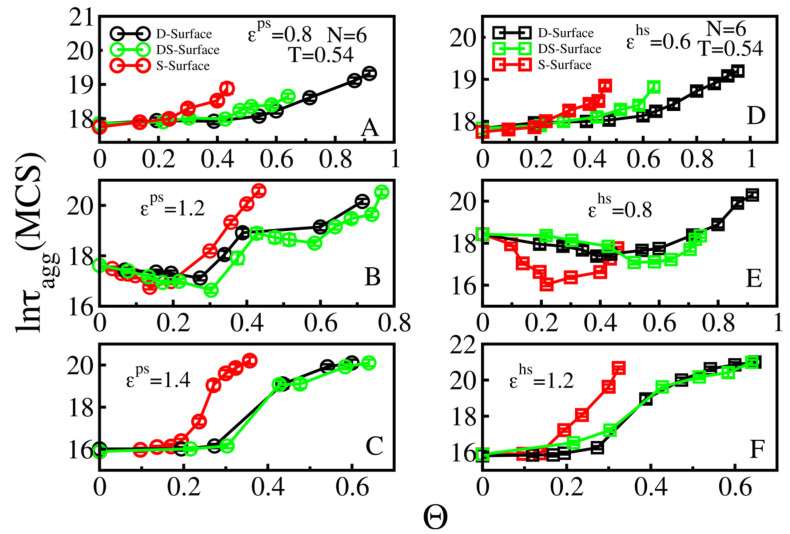
Dependence of lnτ_agg_ on Θ for N = 6 chains at T = 0.54. Red, green and black circles refer to the S-, DS-, D-surfaces, respectively. Results are shown for the weakly ε^ps^ = 0.8 (**A**), medium ε^ps^ = 1.2 (**B**), strongly ε^ps^ = 1.4 (**C**) absorbing hydrophilic surfaces, as well as for weak ε^hs^ = 0.6 (**D**), medium ε^hs^ = 0.8 (**E**) and strong ε^hs^ = 1.2. (**F**) regimes of attractive hydrophobic surfaces. Error bars are lower than data symbols.

As we can see in Figure 6, the dependence of lnτ_agg_ on roughness Θ for weakly, moderately, strongly absorbing hydrophilic and hydrophobic of S-surfaces remain the same for DS- and D-surfaces. This result implied that despite the difference in roughness nature, the dependence of the aggregation time on the roughness is driven a general principle, which depends on the interplay between energetic and entropic factors. For weakly (entropy driven, Figure 6A,D) and strongly (energy driven, Figure 6C,F) absorbing surfaces, the dependence of τ_agg_ on Θ is monotonic, while for the medium case (Figure 6B,E) the U-shape dependence occurs due to the competition between these two factors. Overall, our results agree with the experiment [29], which showed that at a high enough roughness, a surface can block the self-assemble process.

### 3.4. Size Effects

#### 3.4.1. Smooth Surfaces

**Figure 7:**
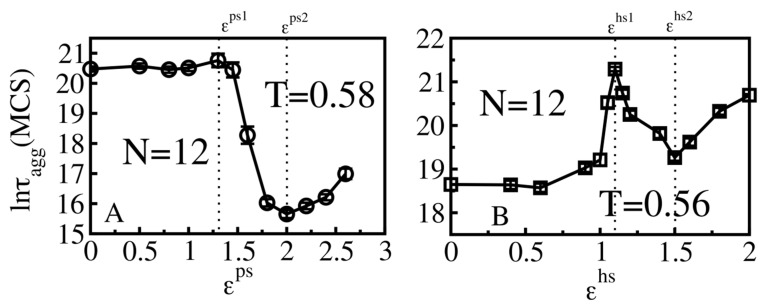
Effect of various surfaces on the aggregation time of N = 12 polypeptide chains at T = 0.58 for smooth hydrophilic surfaces (**A**) and at T = 0.56 for hydrophobic surfaces (**B**). The dash lines at values ε^ps1^ = 1.3, ε^ps2^ = 2.0, ε^hs1^ = 1.1 and ε^hs2^ = 1.5 separate the different interaction ranges corresponding to different types of classified surfaces.

To examine the size effect, we extended simulations to a system of N = 12 peptides (Figure 7). For smooth surfaces, the dependence of the aggregation rate on the protein–surface interaction is similar to the N = 6 case (Figure 4). At T = 0.58 (Figure 7A), for attractive hydrophilic surfaces, no clear effect was seen for ε^ps^ < ε^ps1^ = 1.3, however, the moderately absorbing surface (1.3 < ε^ps^ < 2.0) remarkably accelerates the aggregation process, as ln*τ*_agg_ decreases from 20.5 to 15.65. The retardation by strongly attractive hydrophilic surfaces appears at ε^ps^ > 2.0. The same scenarios occur for hydrophobic smooth surfaces (Figure 7B), however, at T = 0.56, weakly attractive hydrophobic smooth surfaces (ε^hs^ < ε^hs1^ = 1.1) noticeably decreased the peptide self-assembly while the catalyzing effect of a medium absorbing smooth surface happened for 1.1 ≤ ε^hs^ ≤ 1.5. The strongly attractive surfaces with ε^hs^ > ε^hs2^ = 1.5 restricted the formation of fibril-like structure of peptides.

#### 3.4.2. Rough Surfaces

**Figure 8:**
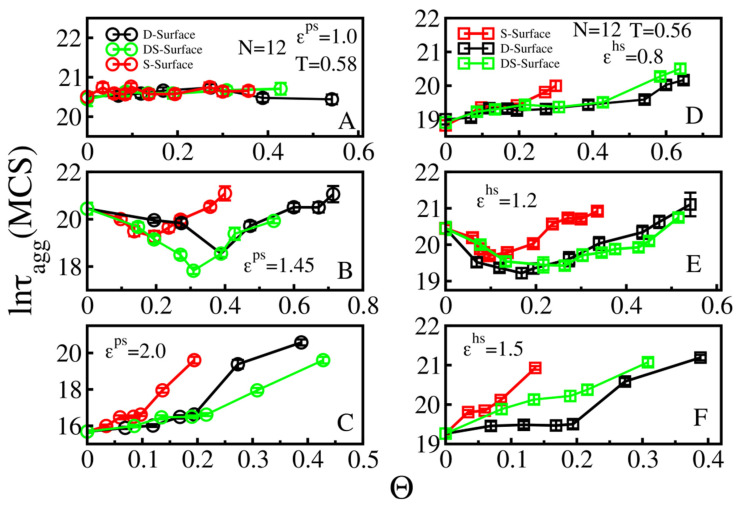
Dependence of lnτ_agg_ on Θ of N = 12 peptides at T = 0.58 and 0.56 for hydrophilic (**left column**) and hydrophobic surfaces (**right column**). For hydrophilic surfaces, we considered ε^ps^ = 1.0 (weak regime, **A**), 1.45 (medium, **B**), and 2.0 (strong, **C**). For hydrophobic surfaces the corresponding values ε^hs^ = 0.8 (**D**), 1.2 (**E**), and 1.5 (**F**).

As in the smooth surface case, for N = 12 peptides, we also systematically investigated the effect of roughness on the aggregation time in the weak, medium and strong peptide–surface interaction regimes for both hydrophilic and hydrophobic surfaces. Qualitatively, the results are similar to the N = 6 case (compare Figure 6 and Figure 8). However, for ε^ps^ = 1 (Figure 8A) the Θ dependence is rather weak and this is probably because the aggregation mainly occurs in the bulk due to the weak interaction between polypeptide chains and the hydrophilic surface. The surface effect is more pronounced in the case of hydrophobic surface with ε^hs^ = 0.8, as the aggregation time increases with increasing Θ (Figure 8D). The U-shape appears in the medium regime (Figure 8B,E), and this effect is stronger in the presence of D- and DS- surfaces. The highly attractive rough surfaces significantly increased the aggregation time and can even inhibit the process at Θ > 0.42 (Figure 8C) and Θ > 0.38 (Figure 8F) for the hydrophilic and hydrophobic cases, respectively.

Thus, the similar behavior of the N = 6 and N = 12 systems with smooth and rough surfaces of various types indicates that our results should be valid for larger systems.

## 4. Conclusions

Since protein aggregation, which is associated with neurodegenerative diseases, occurs in vivo, understanding the influence of various surfaces on this process plays a crucial role in developing new effective therapies. Furthermore, knowledge of the self-assembly mechanism on surfaces is also useful for developing novel materials of fibrillar structure. We constructed a simple lattice model that enabled us to theoretically access the effect of surface roughness on the aggregation kinetics of polypeptide chains. Our model is reliable as it can capture the experimental results of Robert Vacha et al. [35] obtained for both hydrophobic and hydrophilic smooth surfaces that the dependence of the aggregation time on the protein–surface interaction is nontrivial. In addition, our model can explain the experiment of Shezal et al. [29], showing that a rough surface retards the fibril formation and even block it at a high roughness level [48].

By a systematic investigation of the dependence of fibril formation time on the roughness of different surfaces, for the first time, we predicted the U-shape behavior, claiming that a rough surface can not only slow down the aggregation process, but can also accelerate it at a suitable degree of roughness. This can occur in a regime where the entropic factor competes with the energetic factor. Our results were obtained using simple models, but they should be applied to more complex systems, because their validity is guaranteed by general principles, but not by some details. This conclusion is partially supported by the fact that our modeling captures the slowing down or acceleration of aggregation on different surfaces such as lipid membranes [13,14,15,16], mica and glass [17,18], carbon nanotubes [21,22], nanoparticles [23,24], HOPG plane [25,26], etc. From this point of view, more advanced off-lattice models should not qualitatively change these results.

Our study also pointed out that the dependence of aggregation time on surface characteristics is complicated, not only by roughness, but also by the geometry of objects that make the surface non-smooth. It would be interesting to experimentally verify our prediction of the U-shaped dependence of aggregation time on roughness.

For the same sequence, the polypeptide chains form different fibrillary structures on the hydrophobic and hydrophilic surfaces (Figure 2). In particular, on a hydrophobic surface, the fibril-prone structure N* has the same structure as the native monomeric structure, which suggests that the hydrophobic surface alters the morphology of fibrils to a greater extent than the hydrophilic surface. This conclusion could be verified by other more advanced theoretical models and experiments.

## Figures and Tables

**Table 1:** Interaction energies between two beads of polypeptide chains *e_ij_* in the lattice model. The energy is measured in *ε*_H_, where *ε*_H_ is the hydrogen bond energy.

Beads	H	P	+	−
**H**	−1.0	0.2	0.2	0.2
**P**	0.2	−0.2	−0.2	−0.2
+	0.2	−0.2	0.35	−0.7
−	0.2	−0.2	−0.7	0.35

**Table 2:** Interaction energies between the polypeptide chain (H, P, +, and −) and beads of hydrophilic (Ps) and hydrophobic (Hs) surfaces. ε^ps^ and ε^hs^ can be tuned in simulation.

Beads/Balls	H	P	+	−
Ps	0.2	−ε^ps^	−0.2	−0.2
Hs	−ε^hs^	0.2	0.2	0.2

## Data Availability

Our homemade code for MC simulations and the data that support the findings of this study are available from the corresponding author upon reasonable request.
